# The Impact of Selective Spatial Attention on Auditory–Tactile Integration: An Event-Related Potential Study

**DOI:** 10.3390/brainsci14121258

**Published:** 2024-12-15

**Authors:** Weichao An, Nan Zhang, Shengnan Li, Yinghua Yu, Jinglong Wu, Jiajia Yang

**Affiliations:** Graduate School of Interdisciplinary Science and Engineering in Health Systems, Okayama University, 3-1-1 Tsushima-Naka, Okayama 700-8530, Japan; pquy1ha9@s.okayama-u.ac.jp (W.A.);

**Keywords:** auditory–tactile integration, selective spatial attention, event-related potential, left–right hemispace differences, spatiotemporal distribution

## Abstract

Background: Auditory–tactile integration is an important research area in multisensory integration. Especially in special environments (e.g., traffic noise and complex work environments), auditory–tactile integration is crucial for human response and decision making. We investigated the influence of attention on the temporal course and spatial distribution of auditory–tactile integration. Methods: Participants received auditory stimuli alone, tactile stimuli alone, and simultaneous auditory and tactile stimuli, which were randomly presented on the left or right side. For each block, participants attended to all stimuli on the designated side and detected uncommon target stimuli while ignoring all stimuli on the other side. Event-related potentials (ERPs) were recorded via 64 scalp electrodes. Integration was quantified by comparing the response to the combined stimulus to the sum of the responses to the auditory and tactile stimuli presented separately. Results: The results demonstrated that compared to the unattended condition, integration occurred earlier and involved more brain regions in the attended condition when the stimulus was presented in the left hemispace. The unattended condition involved a more extensive range of brain regions and occurred earlier than the attended condition when the stimulus was presented in the right hemispace. Conclusions: Attention can modulate auditory–tactile integration and show systematic differences between the left and right hemispaces. These findings contribute to the understanding of the mechanisms of auditory–tactile information processing in the human brain.

## 1. Introduction

Multisensory integration means that information from different modalities (e.g., vision, hearing, touch, etc.) is merged in the brain to produce a more comprehensive perceptual experience [1,2,3,4]. This process plays a crucial role in increasing perceptual accuracy, reaction speed, and environmental adaptability [5,6]. Auditory–tactile integration, as an important sensory combination method, has received increasing attention in recent years in the study of multisensory integration [7,8,9,10]. Understanding the neural mechanisms of auditory–tactile integration is important for revealing the complexity of human perception and cognitive functions.

Attention is a critical cognitive function that regulates sensory information processing, and attention has a significant effect on the efficiency and effectiveness of multisensory integration [11,12]. Attention can increase the processing of relevant sensory information, inhibit irrelevant inputs, and increase perceptual accuracy and response speed [13,14,15]. The temporal dynamics and neural mechanisms of attention in multisensory integration have been further explored in recent studies. For example, Ferrari and Noppeney [16] reported that attention can increase multisensory integration at early sensory processing stages. Elshout et al. [17] investigated the synergistic effect of motion coherence and multisensory integration and reported that visual–auditory integration can significantly increase information processing in the premotor-related visual task stage, indicating that attention has a beneficial effect on the process of multisensory integration. Selective spatial attention refers to the ability to focus attention on specific locations in space based on task instructions or cues [13]. Previous research has demonstrated that the audiovisual integration effect is stronger at the attended location relative to an unattended location [18]. In addition, other studies have shown that multisensory integration may occur automatically in some cases and is not entirely controlled by attention [19]. This automatic integration process may be more obvious when the stimulus intensity is high, the task load is low, or the stimuli are highly consistent in time and space [20,21]. At present, most research has focused on the effect of attention on audiovisual integration, and it is still unclear how auditory–tactile integration is regulated by attention.

The temporal dynamics of multisensory integration involve different neural processing stages that may occur in the ipsilateral and contralateral hemispheres of the brain [21,22]. The early stages of integration usually take place in the primary sensory cortex and are more related to stimulation of the contralateral hemisphere [23]. For example, Foxe et al. [24] reported that the early components of audiovisual integration occur mainly in the contralateral hemisphere to the side on which the stimulus is presented. The later stages of integration involve more cognitive regions, possibly extending across both hemispheres [25,26]. Molholm et al. [27] reported that the later stages of audiovisual integration were significantly represented in both hemispheres, suggesting that multisensory information is integrated across a wide range of brain regions during the cognitive processing stage. For auditory–tactile integration, Murray et al. [28] found through event-related potential (ERP) research that the early component (approximately 50 ms) of auditory–tactile integration mainly occurs in the hemisphere corresponding to the side on which the stimulus is presented, reflecting the rapid response of primary sensory areas to multisensory information. Gobbelé et al. [29] reported that between 75–85 ms and 105–130 ms, somatosensory stimulation is located in the posterior parietal cortex and between the secondary somatosensory cortex and auditory cortex, respectively. Brett-Green et al. [30] reported that, in an ERP study, significant multisensory integration occurred between 60 and 80 ms in the hemisphere opposite that of the somatosensory stimulation and between 110 and 150 ms in the hemisphere ipsilateral to the somatosensory stimulation. Between 180 and 220 ms, significant multisensory integration occurred in both hemispheres. This finding suggests that the integration process may involve complex neural networks and spatiotemporal characteristics, and whether this process can be regulated by attention and how it is regulated by attention remain unclear. Therefore, further research on the hemispheric involvement of auditory–tactile integration at different temporal stages under different attention conditions is required.

Although previous research has revealed the effects of attention and spatial location on multisensory integration, the following issues still need to be addressed in the field of auditory–tactile integration: (1) Current research has demonstrated insufficient understanding of the mechanism of attention in auditory–tactile integration, especially the differences in the temporal dynamics of integration under different attention conditions and how these processes are modulated by the spatial location of stimulus presentation. (2) Further research is needed to investigate how auditory–tactile integration occurs in the ipsilateral and contralateral hemispheres at different time stages and how these processes are modulated by attention and spatial location. To this end, we designed an ERP experiment during which a series of rapid auditory, tactile, or auditory–tactile stimuli were randomly presented with equal probability to either the left hemispace or the right hemispace. In each block, participants attended to the auditory, tactile, and auditory–tactile stimuli on the designated side and ignored all stimuli on the other side. The participant’s task was to detect target stimuli that were uncommon on the attended side and to report these stimuli as quickly and accurately as possible via a foot pedal. The effect of attention on the neural mechanisms of auditory–tactile integration was revealed by analyzing ERP data from four time windows (70–90 ms, 90–110 ms, 110–130 ms, and 180–220 ms) and seven electrodes of interest (central-parietal: AFz, FCz, and CPz; lateral: C3, C4, CP5, and CP6).

## 2. Materials and Methods

### 2.1. Participants

Twenty participants (11 males, aged 18–34 years, mean age = 24.2 years) were included in this study. All the participants had normal hearing and tactile senses and reported having a right-hand preference. No participants reported a history of neurological disorders. All the participants signed an informed consent form prior to the experiment. The participants received monetary compensation for taking part in the experiment. This study was approved by the local ethics committee.

### 2.2. Stimuli

**Figure 1:**
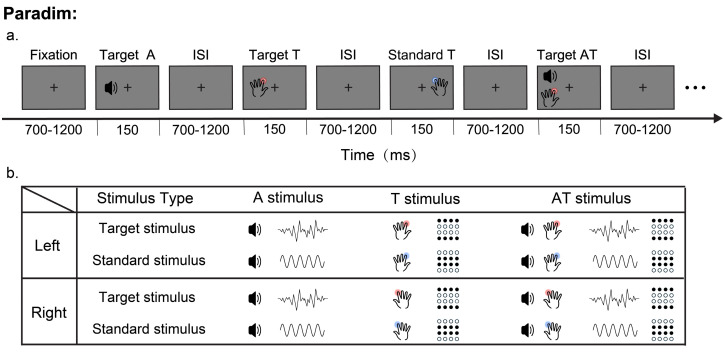
Experimental paradigm. (**a**) Sequence of events and their duration. (**b**) Stimulus conditions: There were 12 stimulus conditions in total: 8 unisensory stimuli (4 standard stimuli and 4 target stimuli) and 4 multisensory stimuli (2 standard stimuli and 2 target stimuli). The black dots in the tactile stimulus column indicate that the probe was raised.

Tactile (T) and auditory (A) stimuli were presented during the task. The stimuli were divided into standard stimuli and target stimuli. The T stimuli were delivered to the fingertips of the left and right index fingers of the participant via a customized piezoelectric braille display (Piezostimulator, SC9 device, KGS Co., Ogawa, Ogawa-cho, Hiki-gun, Saitama, Japan). As shown in Figure 1, the standard T stimuli were the second and third rows of raised probes, and the target stimuli were the first and fourth rows of raised probes. The A stimuli were delivered to the participant’s left and right ears through soundproof headphones. The standard stimulus was a pure tone of a 1000 Hz sine wave, and the target stimulus was white noise. The loudness of both the standard stimulus and the target stimulus was 60 dB (a).

### 2.3. Procedure

The experiment was conducted in a dimly lit room with sound attenuation and electrical shielding. Each participant completed 10 blocks, with each block containing 80 A, 80 T, and 80 auditory–tactile (AT) stimuli. Each modality stimulus was divided into 20% (16) target stimulus and 80% (64) standard stimulus. Each kind of stimulus (2 (standard and target) × 3 (A, T, and AT)) was presented in pseudorandom order with equal probability to the left and right of the participant. The duration of the stimuli was 150 ms, and the interstimulus intervals were randomized between 700 and 1200 ms to avoid anticipation effects.

The participants were seated in a comfortable chair with their head placed in a chin rest for head position fixation. Their hands were naturally placed on the table with the forearms perpendicular to the body plane and 70 cm between the left and right hands. Before the start of each block, the participants were instructed to attend to all the stimuli presented on designated side (left or right) and to detect infrequently occurring target stimuli while ignoring all the stimuli on the other side. The participants were asked to complete several practice blocks to understand the task. To avoid motion artifacts, the participants were also instructed to minimize blinking and body movements. During the experiment, participants were required to fixate on the centrally presented fixation point. The participants were instructed to press the pedal with their right foot as quickly and accurately as possible when they heard and/or felt the target stimulus. The participants were required to attend to all the stimuli on the left side in five blocks and to attend to all the stimuli on the right side in another five blocks. The two types of blocks alternated. The participants were free to rest between blocks.

### 2.4. Electroencephalographic Data Recording and Processing

Encephalographic (EEG) data were recorded via a 64-channel EEG cap (Easy-cap, Wörthsee, Germany) and a BrainAmp DC amplifier (Gilching, Germany). Horizontal eye movements were recorded from an electrode placed at the outer canthus of the left eye, and vertical eye movements were recorded from an electrode placed below the right eye. The FCz electrode was used as the online reference, with the electrode impedance maintained at 5 kΩ and a sampling rate of 1000 Hz.

EEG data were preprocessed in MATLAB R2021b via EEGLAB (version 2024.0), and offline analysis was used to re-reference all electrodes to the average value of the bilateral mastoid. The EEG data were bandpass filtered in the range of 0.01–30 Hz, and then the continuous EEG data were segmented (−100~500 ms). Baseline corrections were made from −100 ms to 0 ms. Independent component analysis was then performed to remove noise interference, such as that from eye and muscle movements. Artifacts were further corrected by suppressing signals exceeding ±80 μV.

### 2.5. Data Analyses

The effect of multisensory integration was assessed by comparing the responses to multisensory stimuli with the algebraic sum of the responses to unisensory stimuli. If there was a significant difference between the sum of the ERPs to unisensory stimuli and the ERP to multisensory stimuli, then multisensory interaction was said to have occurred [31,32].

**Figure 2:**
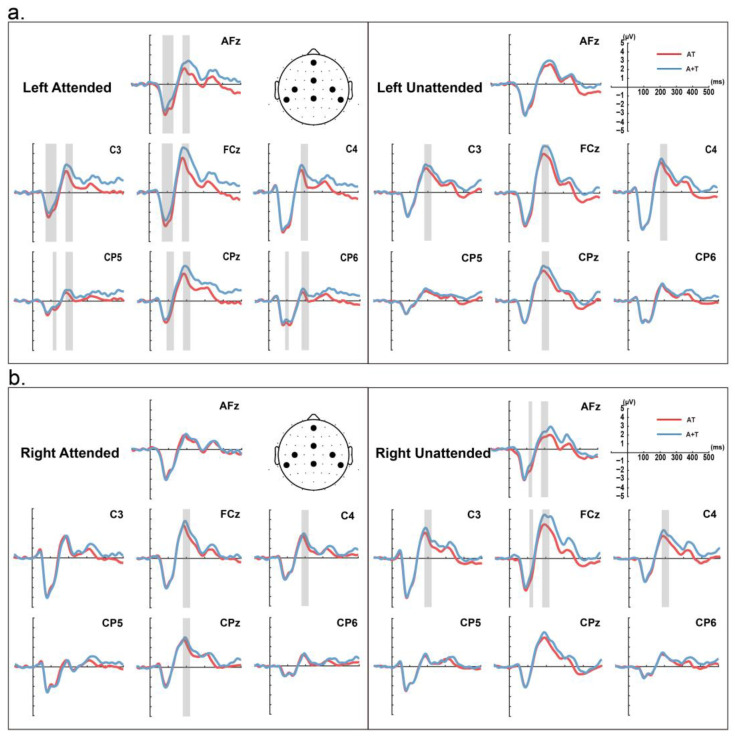
Event-related potentials (ERPs) of standard stimulus. The figure shows the grand average of ERPs for unisensory stimulus summation (blue trace) and simultaneous auditory and somatosensory stimulation (red trace) in 20 participants. ERPs are shown at central electrode positions (AFz, FCz, and CPz) and lateral electrode positions (C3, CP5, C4, and CP6). (**a**) Stimulus presented in the left half-space; (**b**) stimulus presented in the right half-space. The shaded gray part indicates a significant difference in stimulus type.

**Figure 3:**
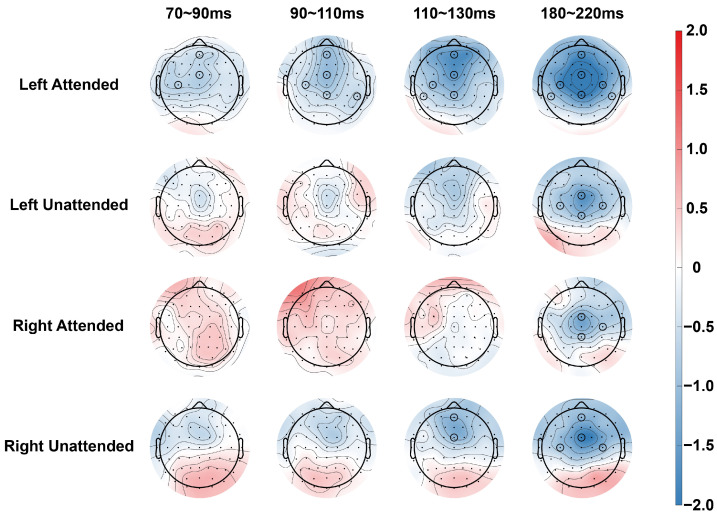
Influence of attention on topographic voltage distribution. The topographic voltage distributions of the grand average event-related potential (ERP) components for the standard stimulus in the 4 time windows of interest for attended and unattended stimuli when the stimuli were presented in the left and right hemispaces, respectively. The maps show the mean voltage of AT − (A + T) within the corresponding time windows (70–90 ms, 90–110 ms, 110–130 ms, and 180–220 ms). A, auditory; T, tactile; AT, auditory–tactile. Black circles indicate electrodes with significant differences in stimulus type.

For ERP analysis, as shown in Figure 2 and Figure 3, the average amplitude of the neural response to the standard stimulus was calculated for each stimulus condition within selected time windows. On the basis of previous studies and our results, we selected four time windows (N80: 70~90 ms, P100: 90~110 ms, N100: 110~130 ms, and P200: 180~220 ms) and seven electrodes (AFz, FCz, CPz, C3, CP5, C4, and CP6) for further analysis [30,33].

To examine multisensory integration under different conditions, a three-way repeated-measures analysis of variance (ANOVA) was first performed for the central electrodes, separately for stimuli presented on the left and right sides, with the following factors: attention (attended vs. unattended), stimulus type (AT vs. A + T), and electrode position (AFz, FCz, and CPz). In order to examine the auditory–tactile integration in the contralateral and ipsilateral hemispheres under different conditions, a within-subject 3-way repeated measures ANOVA was performed on the two pairs of contralateral electrodes (C3/4, CP5/6, the factors included attention (attended vs. unattended), stimulus type (AT vs. A + T), and hemisphere (same side as the stimuli vs. opposite side of the stimuli). When the spherical assumptions were violated, corrections were made using the Greenhouse–Geisser correction, and multiple comparison corrections were made using the Bonferroni correction. We report only statistically significant effects that included the channel type factor.

## 3. Results

### 3.1. N80 (70~90 ms)

When stimuli were presented in the left hemispace, an ANOVA for the central electrode showed a significant main effect of stimulus type [F(1,19) = 4.493, *p* = 0.047, *η^2^p* = 0.191], and subsequent comparisons (paired *t*-tests) found that there was a significant difference of stimulus type on the two electrodes under attended conditions (AFz [t(19) = 2.964, *p* = 0.008, Cohen’s d = 0.313], FCz [t(19) = 2.460, *p* = 0.024, Cohen’s d = 0.395]), and no significant difference of stimulus type was found in the unattended condition. For the lateral electrodes, ANOVA revealed no significant main effects or interactions for stimulus type, and subsequent comparisons revealed a significant difference of stimulus type in the ipsilateral hemisphere in the attended condition (C3 [t(19) = 2.628, *p* = 0.017, Cohen’s d = 0.454]). No significant difference of stimulus type was found in the unattended condition.

For stimuli in the right hemispace, the ANOVA for central electrodes revealed a significant interaction between attention and stimulus type [F(1,19) = 4.418, *p* = 0.049, *η^2^p* = 0.189] and a significant interaction between stimulus type and electrode position [F(1,19) = 4.939, *p* = 0.021, *η^2^p* = 0.206], but subsequent comparisons did not reveal any significant differences related to stimulus type. ANOVA and subsequent comparisons for lateral electrodes did not reveal significant differences in stimulus type. No significant differences in stimulus type were found via ANOVA or subsequent comparisons for the lateral electrodes.

### 3.2. P100 (90~110 ms)

For stimuli in the left hemispace, an ANOVA on the central electrode revealed a significant main effect of stimulus type [F(1,19) = 5.293, *p* = 0.033, *η^2^p* = 0.218] and a significant interaction between stimulus type and attention [F(1,19) = 5.033, *p* = 0.037, *η^2^p* = 0.209]. Subsequent comparisons revealed significant differences of stimulus type at three electrodes in the attended condition (AFz [t(19) = 4.492, *p* < 0.001, Cohen’s d = 0.469], FCz [t(19) = 4.580, *p* < 0.001, Cohen’s d = 0.504] and CPz [t(19) = 2.482, *p* = 0.025, Cohen’s d = 0.333]). No significant difference of stimulus type was found in the non-attended condition. For the lateral electrodes, ANOVAs found significant interactions between attention and stimulus type [F(1,19) = 5.917, *p* = 0.025, *η^2^p* = 0.237] on the C3/4 electrode pair, as well as significant interactions between attention and stimulus type [F(1,19) = 4.811, *p* = 0.041, *η^2^p* = 0.202] on the CP5/6 electrode pair. Subsequent comparisons revealed that significant differences of stimulus type were found in both hemispheres in the attended condition (C3 [t(19) = 3.312, *p* = 0.004, Cohen’s d = 0.414], CP6 [t(19) = 2.571, *p* = 0.019, Cohen’s d = 0.449]). No significant differences in channel type were found in the unattended condition.

For stimuli in the right hemispace, neither ANOVA nor subsequent comparisons of central and lateral electrodes revealed significant differences of stimulus type between attended and unattended conditions.

### 3.3. N100 (110~130 ms)

When stimuli were presented in the left hemispace, the ANOVA for the central electrode showed a significant main effect of stimulus type [F(1,19) = 12.122, *p* = 0.002, *η^2^p* = 0.390] and a significant interaction between attention and stimulus type [F(1,19) = 5.297, *p* = 0.033, *η^2^p* = 0.217]. Subsequent comparisons revealed significant differences of stimulus type for the three electrodes in the attended condition (AFz [t(19) = 5.486, *p* < 0.001, Cohen’s d = 0.673], FCz [t(19) = 4.631, *p* < 0.001, Cohen’s d = 0.660] and CPz [t(19) = 2.753, *p* = 0.013, Cohen’s d = 0.448]). For the lateral electrodes, ANOVA revealed a significant interaction between stimulus type and electrode position [F(1,19) = 4.850, *p* = 0.040, *η^2^p* = 0.203] on the C3/4 electrode pairs, and no main effect or interaction of stimulus type was found on the CP5/6 electrode pairs. Subsequent comparisons revealed significant differences of stimulus type in the ipsilateral hemisphere for the attended condition (C3 [t(19) = 3.614, *p* = 0.002, Cohen’s d = 0.491] and CP5 [t(19) = 2.456, *p* = 0.024, Cohen’s d = 0.283]).

When stimuli were presented in the right hemispace, ANOVA for central electrodes revealed a significant interaction between attention and stimulus type and electrode position [F(1.578,29.983) = 4.625, *p* = 0.025, *η^2^p* = 0.196]. Simple effects analyses revealed a significant difference of stimulus type on the two electrodes in the unattended condition (AFz [t(19) = 2.714, *p* = 0.014, Cohen’s d = 0.438] and FCz [t(19) = 2.379, *p* = 0.028, Cohen’s d= 0.475]). Neither ANOVA nor subsequent comparisons for lateral electrodes revealed significant differences of stimulus type in any condition.

### 3.4. P200 (180~220 ms)

When stimuli were presented in the left hemispace, an ANOVA for the central electrode showed a significant main effect of stimulus type [F(1,19) = 24.342, *p* < 0.001, *η^2^p* = 0.562], a significant interaction between stimulus type and attention [F(1,19) = 5.942, *p* = 0.025, *η^2^p* = 0.238], and a significant interaction between stimulus type and electrode position [F(1.906,36.212) = 9.627, *p* < 0.001, *η^2^p* = 0.336]. Subsequent comparisons revealed significant differences of stimulus type at three electrodes in the attended condition (AFz [t(19) = 3.578, *p* = 0.002, Cohen’s d = 0.532], FCz [t(19) = 5.850, *p* < 0.001, Cohen’s d = 0.912] and CPz [t(19) = 6.004, *p* < 0.001, Cohen’s d = 0.666]), and a significant difference of stimulus type at the two electrodes in the unattended condition (FCz [t(19) = 2.989, *p* = 0.008, Cohen’s d = 0.514] and CPz [t (19) = 2.817, *p* = 0.011, Cohen’s d = 0.342]). For the lateral electrodes, the ANOVA showed a significant main effect of stimulus type [F(1,19) = 19.368, *p* < 0.001, *η^2^p* = 0.505] and a significant interaction between stimulus type and attention [F(1,19) = 4.741, *p* = 0.042, *η^2^p* = 0.200] on the C3/4 electrode pairs. The main effect of stimulus type [F(1,19) = 12.037, *p* = 0.003, *η^2^p* = 0.338] was significant on the CP5/6 electrode pair. Subsequent comparisons revealed significant differences of stimulus type in both hemispheres in the attended condition (C3 [t(19) = 5.981, *p* < 0.001, Cohen’s d = 0.819], C4 [t(19) = 4.170, *p* < 0.001, Cohen’s d = 0.736], and CP5 [t(19) = 4.483, *p* < 0.001, Cohen’s d = 0.495] and CP6 [t(19) = 2.908, *p* = 0.009, Cohen’s d = 0.499]), significant differences of stimulus type were also found in both hemispheres under the unattended condition (C3 [t(19) = 2.427, *p* = 0.025, Cohen’s d = 0.387], C4 [t(19) = 2.305, *p* = 0.033, Cohen’s d = 0.429]).

When stimuli were presented in the right hemispace, ANOVA results for the central electrode showed a significant main effect of stimulus type [F(1,19) = 8.922, *p* = 0.008, *η^2^p* = 0. 0.320], and a significant interaction between stimulus type and electrode location [F(1,19) = 9.432, *p* < 0.001, *η^2^p* = 0.332]. Subsequent comparisons revealed significant differences of stimulus type at two electrodes in the attended condition (FCz [t(19) = 3.281, *p* = 0.004, Cohen’s d = 0.471] and CPz [t(19) = 2.795, *p* = 0.012, Cohen’s d = 0.278]) and a significant difference of stimulus type at the two electrodes in the unattended condition (AFz [t(19) = 2.223, *p* = 0.039, Cohen’s d = 0.374] and FCz [t(19) = 3.013, *p* = 0.007, Cohen’s d = 0.691]). ANOVA of the lateral electrodes revealed a significant main effect of stimulus type [F(1,19) = 9.844, *p* = 0.005, *η^2^p* = 0.341] on the C3/4 electrode pair. Subsequent comparisons revealed significant differences of stimulus type in the ipsilateral hemisphere in the attended condition (C4 [t(19) = 2.439, *p* = 0.025, Cohen’s d = 0.386]) and in the bilateral hemisphere in the unattended condition (C3 [t(19) = 2.723, *p* = 0.013, Cohen’s d = 0.470], C4 [t(19) = 2.538, *p* = 0.020, Cohen’s d = 0.511]).

## 4. Discussions

In this study, ERP technology was used to explore the effect of spatially selective attention on auditory–tactile integration. The ERP experimental results showed that for stimuli presented in the left hemispace, auditory–tactile integration occurred earlier and had a wider spatial range in the attended condition. For stimuli presented in the right hemispace, integration occurred later, and integration occurred in a wider temporal and spatial range in the unattended condition. In addition, the difference in integration between the attended and unattended conditions was not as obvious as when the stimuli were presented on the left side.

When stimuli were presented in the left hemispace, the results showed that there were more brain regions where auditory–tactile integration occurred under the attended condition, and the integration effect appeared earlier. Specifically, in the attended condition, integration was observed at central electrodes AFz and FCz within an early time window of N80, and the extent of integration gradually expanded over time in the subsequent time windows. This finding is consistent with previous studies and indicates that attention can promote multisensory integration through the collaborative processing of related sensory information [12,13]. The early integration effect suggests that attention contributes to multisensory integration at the primary sensory processing stage [34]. For example, Talma et al. found that spatial selective attention affects the early P100 component, and the integration effect is greater under attended conditions. P100 associates with early sensory processing and primary attention allocation, affecting primary sensory filtering and perceptual enhancement [35,36]. N100 (or N1) is generally considered an indicator of selective attention at the perceptual stage and is associated with enhanced discrimination and orientation to the features and location of stimuli [37]. P200 (or P2) generally relates to mid- or late-stage cognitive processing, including stimulus categorization, semantic integration, and higher-level perceptual decision making [38]. This phenomenon may be due to the fact that attention increases the excitability of the sensory cortex and increases sensitivity to multisensory stimuli [11]. In addition, frontal regions are involved in attentional control and higher cognitive functions [39], and the activation of frontal regions suggests that attention may regulate the early processing of auditory–tactile integration through frontal regions [40]. For the lateral electrode analysis, significant integration was found at N80 and N100 on the side of the brain ipsilateral of the stimulus and at P200 on both sides of the brain; these findings are consistent with previous research results [29,30]. In the time window of P100, previous studies have revealed multisensory integration only in the ipsilateral hemisphere [41]. Our results revealed significant integration in the contralateral hemisphere, which may be due to the addition of attentional resources.

Conversely, when the stimuli were presented in the right hemispace, more brain regions were involved in auditory–tactile integration in the unattended condition, and the integration effect mainly appeared in a later time window (N100 and P200). This finding indicates that for stimuli in the right half of space, auditory–tactile integration may be more dependent on automated processing and relatively less affected by attention. This indication is consistent with the view expressed in some studies that multisensory integration may occur automatically in some cases [21,42]. The results of the analysis of the lateral electrodes also revealed the same trend, with later integration shown at the lateral electrodes, which occurred only within a time window of P200. Under the attended condition, integration occurred significantly earlier in the ipsilateral hemisphere than in the contralateral hemisphere, whereas under the unattended condition, integration occurred in both hemispheres. These results suggest that for stimuli in the right hemispace, auditory–tactile integration requires a longer processing time and that the influence of attention is more limited.

Our results showed that spatial location has a significant effect on the temporal dynamics of auditory–tactile integration and the pattern of brain region activation. Attention facilitated auditory–tactile integration when the stimuli were presented in the left hemispace. This finding may be related to the advantage of the right hemisphere in processing stimuli in the left hemispace and spatial attention tasks. The right hemisphere is widely considered to have greater processing capacity for spatial attention and multisensory integration, especially for stimuli in the left hemispace [43,44]. This hemispheric dominance means that when stimuli appear in the left half of space, the right hemisphere can more effectively regulate and integrate information from different sensory modalities, thus achieving a wider integration effect in an earlier time window. In addition, the right hemisphere not only dominates spatial attention, but is also more strongly connected to the integration network of multiple sensory channels [25,45,46]. This highly efficient functional connectivity allows the right hemisphere to quickly and extensively activate relevant brain regions in response to auditory–tactile stimuli in the left half of space, promoting integration effects over a wider range and over a longer period.

## 5. Conclusions

This study revealed that spatially selective attention and the spatial location of stimuli significantly affect the temporal dynamics of auditory–tactile integration and the pattern of brain region activation. Specifically, in the left half of the space, auditory–tactile integration occurred earlier and involved a wider range of brain regions under the attended condition, whereas in the right half of the space, auditory–tactile integration occurred earlier and involved a wider range of brain regions under the unattended condition. In addition, auditory–tactile integration involved the ipsilateral and contralateral hemispheres at different time stages, reflecting the complexity and dynamics of multisensory integration. These findings provide new perspectives for understanding the neural mechanisms of multisensory integration and are highly important for theoretical research and practical applications in related fields.

## Data Availability

The data presented in this study are available upon reasonable request from the corresponding author.
